# Non-homogeneous models of sequence evolution in the Bio++ suite of libraries and programs

**DOI:** 10.1186/1471-2148-8-255

**Published:** 2008-09-22

**Authors:** Julien Dutheil, Bastien Boussau

**Affiliations:** 1 BiRC – Bioinformatics Research Center – University of Aarhus, C. F. Møllers Alle, Building 1110, DK-8000 Århus C, Denmark; 2 UMR CNRS 5558 – Laboratoire de Biométrie et Biologie Évolutive, CNRS, Université de Lyon, Université lyon 1, 43 Boulevard du 11 Novembre, 69622 Villeurbanne, France

## Abstract

**Background:**

Accurately modeling the sequence substitution process is required for the correct estimation of evolutionary parameters, be they phylogenetic relationships, substitution rates or ancestral states; it is also crucial to simulate realistic data sets. Such simulation procedures are needed to estimate the null-distribution of complex statistics, an approach referred to as parametric bootstrapping, and are also used to test the quality of phylogenetic reconstruction programs. It has often been observed that homologous sequences can vary widely in their nucleotide or amino-acid compositions, revealing that sequence evolution has changed importantly among lineages, and may therefore be most appropriately approached through non-homogeneous models. Several programs implementing such models have been developed, but they are limited in their possibilities: only a few particular models are available for likelihood optimization, and data sets cannot be easily generated using the resulting estimated parameters.

**Results:**

We hereby present a general implementation of non-homogeneous models of substitutions. It is available as dedicated classes in the Bio++ libraries and can hence be used in any C++ program. Two programs that use these classes are also presented. The first one, Bio++ Maximum Likelihood (BppML), estimates parameters of any non-homogeneous model and the second one, Bio++ Sequence Generator (BppSeqGen), simulates the evolution of sequences from these models. These programs allow the user to describe non-homogeneous models through a property file with a simple yet powerful syntax, without any programming required.

**Conclusion:**

We show that the general implementation introduced here can accommodate virtually any type of non-homogeneous models of sequence evolution, including heterotachous ones, while being computer efficient. We furthermore illustrate the use of such general models for parametric bootstrapping, using tests of non-homogeneity applied to an already published ribosomal RNA data set.

## Background

In phylogenetics, simulations have been widely used to study the robustness of inference methods [1] and have been involved in parametric bootstrapping [2]. For instance, simulations have shown that maximum likelihood methods often more accurately reconstructed the evolution of an alignment than distance or parsimony methods [3,4], but could also fail in conditions where compositional biases (a condition here referred to as non-homogeneity) or rate heterogeneity along branches (a phenomenon named heterotachy, [5]) were too intense [6-8]. Similarly, simulations have been used to compare topologies with respect to an alignment [9], or to assess the fit of a model to a particular data set [10-13]. In this last case, a model has a good fit to a particular data set if the alignments it generates have properties similar to the properties of the real alignment. Both for investigating reconstruction methods and for parametric bootstrapping, it is highly desirable that simulation methods model as precisely as possible the conditions that shaped biological sequences through evolution. However, widely-used simulation programs cannot be easily tuned to precisely reproduce the peculiar evolution of a particular data set. Noticeably, non-homogeneity cannot be simulated by Seq-Gen [14] or PAML [15], even if these phenomena are all known to affect the evolution of many data sets [5,16-20].

The ability to estimate parameters of sequence evolution with realistic models, and then computationally evolve sequences using these fitted parameters is crucial to better characterize the behavior of reconstruction methods in realistic settings.

Here we introduce extensions to the Bio++ package [21] that permit first to estimate parameters of evolution on a specific data set in a maximum likelihood framework, and second to simulate the evolution of sequences using these estimated parameters. Importantly, nearly any combination of non-homogeneous (including non-stationary models) and heterotachous models of evolution can be fitted to data, so that simulations may mimic very precisely the evolution of a data set. Such a flexibility should enable one to probe how robust methods of phylogenetic tree or ancestral state reconstruction are to more realistic evolutionary conditions. Moreover, it offers the possibility to compare a large variety of models by assessing through parametric bootstrapping their respective ability to reproduce a given characteristic of interest, measured on a real data set.

## Implementation

Molecular phylogenetic methods are used by a wide range of biologists, from bioinformaticians willing to characterize and improve models of sequence evolution to molecular biologists trying to grasp the particular evolutionary history of their gene of interest. These different types of users have different needs: the former may benefit from easy-to-assemble, high-level object-oriented code to conduct phylogenetic analysis, while the latter likes user-friendly interfaces. However, both demand programs able to run the most recent models of evolution. The newly introduced extensions are available in two flavors that might fit different users' needs: (i) as classes in the Bio++ phylogenetic library, including a special class called SubstitutionModelSet which implements the relationships between models, parameters and branches, and (ii) through the BppML and BppSeqGen programs, which can respectively adjust these models to a data set and simulate data from these models. These programs share a common syntax for model specification and are hence fully inter-operational and easy-to-use.

### The SubstitutionModelSet class

**Figure 1:**
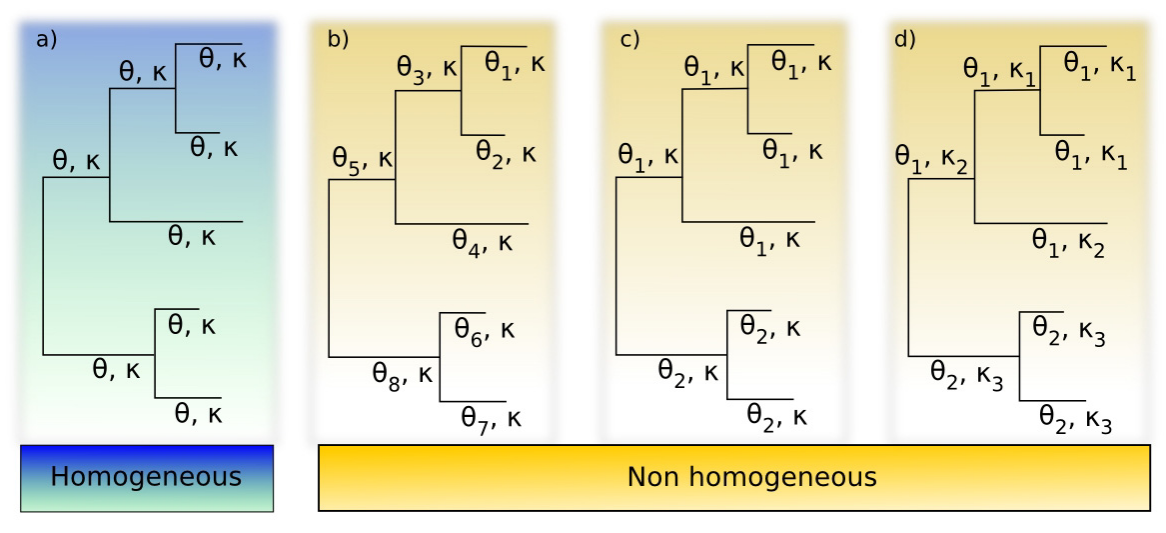
**General non-homogeneous model of substitution**. The substitution model depicted here is Tamura's 1992 model of substitution, which contains two parameters: *κ*, the transitions/transversions ratio and *θ *the equilibrium G+C content. In the homogeneous case, *θ *and *κ *are constant over the tree (case 'a'). In Galtier and Gouy's 1998 model, *κ *is constant over the tree and one distinct *θ *is allowed per branch (case 'b'). Between these two extrema lay models with certain branches, but not all, sharing a common value of *θ *(case 'c'). In the most general case 'd', there are two sets of parameters, one for *κ *and another for *θ*, that are shared by the branches of the tree.

**Figure 2:**
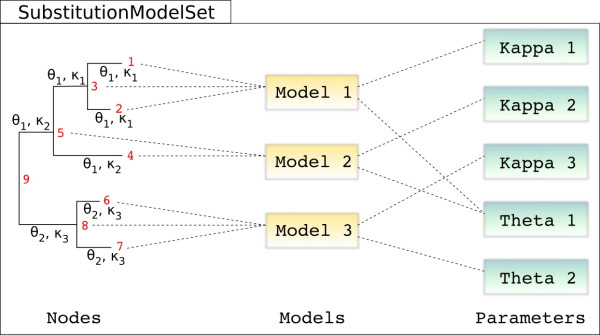
**Relations between branches, models and parameters**. In the general non-homogeneous case, model parameters are shared by different branches across the tree. These parameters are part of branch-specific substitution models, which specify branch-wise probabilities of replacement between states. Branches are here defined according to their rightmost node. The SubstitutionModelSet class stores dependencies between nodes, models and parameters.

The Bio++ libraries [21] provide data structures and algorithms dedicated to analysis of nucleotide, codon and amino acid sequences, phylogenetics and molecular evolution, and are designed in an object-oriented way. These include classes for storing phylogenetic trees, computing likelihood under various models of substitution, and estimating parameters. The likelihood classes take as input a phylogenetic tree and a substitution model, and were extended to allow the computation under non-homogeneous models (figure 1). This support is achieved through the addition of parameters for the rooting of the tree, since the likelihood may not be independent of the root position with a non-homogeneous model [6], and through a new class named SubstitutionModelSet. The SubstitutionModelSet class essentially associates a substitution model with each branch of the phylogenetic tree, and links each substitution model to a list of corresponding parameters (figure 2). It also provides a series of methods for the developer to set up the general model, to assign parameters to substitution models and substitution models to branches.

Substitution models can be totally independent of each other, or can share any number of parameters. Virtually any non-homogeneous model can thus be set up, provided the alignment is not a mix of nucleotide, amino-acid or codon sequences. All models available in Bio++ can be used with this class (*e.g*. K80, T92, HKY85, GTR, JTT92, etc), including heterotachous models (Galtier's model [22] and Tuffley and Steel's model [23]) and any rates across sites model (*i.e*. Gamma and Gamma + invariant distributions). The developer can also use the SubstitutionModelSet class with his own substitution model through the Bio++ SubstitutionModel interface. The SubstitutionModelSet class can be used in conjunction with other Bio++ classes to reconstruct ancestral states or to map substitutions, and hence allows to perform these analyses in the general non-homogeneous case.

### Estimating parameters

Estimation of numerical parameters is performed using a modified Newton-Raphson optimization algorithm, commonly used in phylogenetics [4,24,25], and therefore requires computing derivatives with respect to parameters of the model. Because the use of the cross derivatives leads to numerical instabilities in the optimization (Nicolas Galtier, personal communication), they are set to zero in the Hessian matrix. Derivatives regarding branch lengths are computed analytically, whereas derivatives regarding the rates across sites distribution are computed numerically. Although the substitution model derivatives can be computed analytically in the homogeneous case as well as in Galtier and Gouy's model, they are difficult to compute analytically in the more general case, and are consequently computed numerically in Bio++. To prevent convergence issues due to erroneous derivative values we use, in the last optimization steps, Powell's multi-dimensions algorithm, which does not rely on parameter derivatives [26].

### A general file format to describe non-homogeneous models

We introduced a new user-intuitive property file format to describe non-homogeneous substitution models. This format is an extension of PAML or NHML property file formats, and uses a syntax of the kind

property_name = property_value

**Figure 3:**
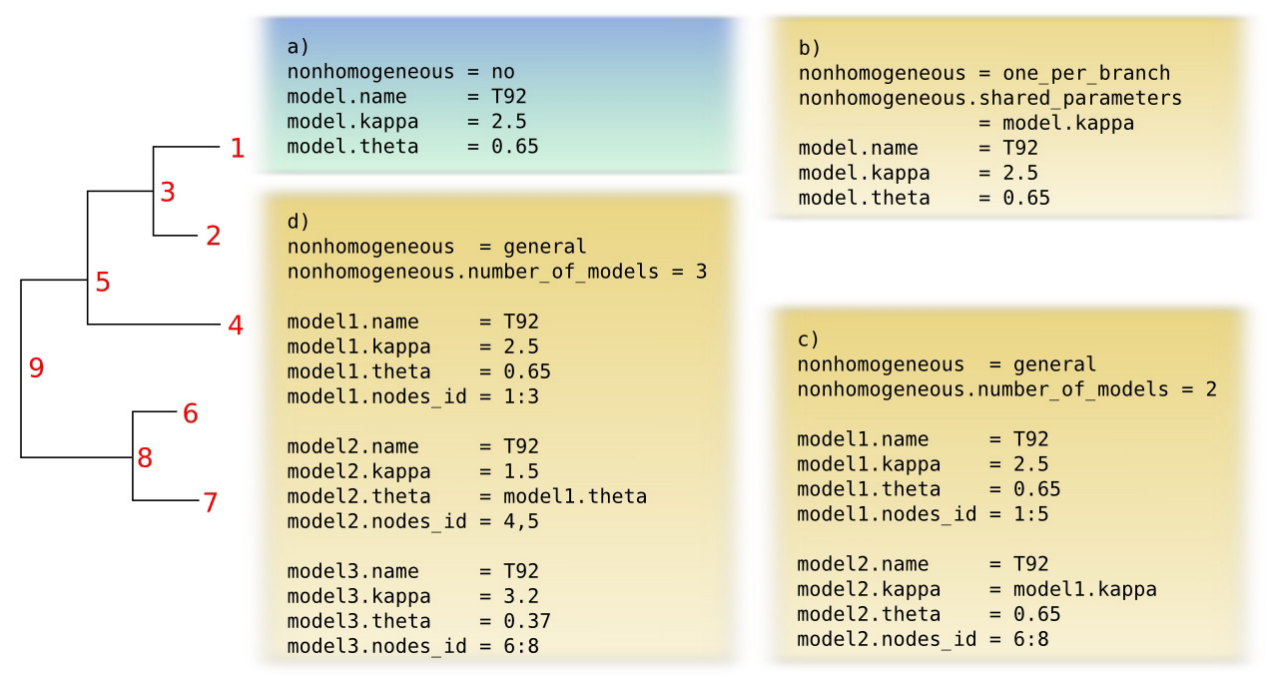
**Model specification in BppML and BppSeqGen**. A general file format is introduced to allow for the user-friendly description of virtually any non-homogeneous model. The tree shows the nodes identifiers, which can be obtained from the programs or defined by the user in its own program. Each case presented here corresponds to a particular model in figure 1, and was labeled accordingly. Each parameter can be fixed to a specific value or optimized with BppML.

A parser that automatically instantiates the appropriate SubstitutionModelSet object is included in the Bio++ libraries and is used by all programs in the Bio++ programs suite. Moreover, the same format is used for the input file of the programs and for their output, so that the output of one program (*e.g*. which adjusts a model to real data) can easily be used as the input of another one (*e.g*. which simulates data from a model). Figure 3 shows how the models in figure 1 are coded using this format. The core part of the description is the "model" property, which is associated to one or several nodes of the phylogenetic tree through node identifiers. These node identifiers can be obtained from the programs in the Bio++ program suite, or set by the user in his own program.

### The BppML and BppSeqGen programs

Parameter estimation and simulation procedures are available as dedicated classes in the Bio++ phylogenetic library, and can hence be used in any C++ program. However, for users who would rely on appropriate software rather than program their own tools, the Bio++ program suite was designed. These programs, including BppML (for Bio++ Maximum Likelihood) and BppSeqGen (Bio++ Sequence Generator) are command line driven and fully parametrized using property files, as introduced above. They can thus easily be pipelined with scripting languages as bash, python or perl. In addition to the BppML and BppSeqGen programs, the Bio++ program suite also contains programs for distance-based phylogenetic reconstruction, sequence file format conversion and tree manipulation.

## Results and Discussion

Our new general non-homogeneous model implementation was applied to Boussau and Gouy's data set of concatenated small and large subunit ribosomal RNA sequences and tree [6]. This data set contains 92 sequences and 527 complete sites. We first compare computation time, memory usage and parameter estimation for various models and software. We then show how the general non-homogeneous model introduced here can be used to study model fit through parametric bootstrapping.

In this section, we use the following model notations:

**H **Homogeneous model, using a Tamura 1992 substitution model [27].

**NH1 **One-theta-per-branch non-homogeneous model [24]. This model uses Tamura's 1992 substitution model, with one *θ *(equilibrium G+C content) per branch in the tree, whereas *κ *(transitions/transversions ratio) is shared by all branches.

**NH2 **One-theta-per-kingdom non-homogeneous model. In this general model, we allowed each kingdom (Bacteria, Eukaryotes or Archaea) to have its own equilibrium G+C content, while sharing the same transitions/transversions ratio.

**NH3 **Same as NH2, but in addition the (hyper)thermophilic Bacteria on one hand, and the eukaryote G+C-rich genus Giardia on the other hand were allowed to have their own equilibrium G+C content.

**NH4 **One-kappa-per-branch non-homogeneous model. This model has one *κ *per branch in the tree, whereas *θ *is shared by all branches.

### Performance

We compared the likelihood of our implementation with the NHML [22,24] and [nh]PhyML [4,6] programs (see table 1 and Additional file 1). Several models have been tested: Kimura two parameters (K80) for the homogeneous case, and Tamura 1992 (T92) derived models for the non-homogeneous cases, with constant rate, Gamma distributed rates (4 classes), Gamma (4 classes) + invariant and Galtier's 2001 site-specific rate variation model (covarion-like). On all tested models, the optimization algorithm in Bio++, while using numerical derivatives, leads to similar or better likelihood values than other programs, although at the price of an increase in computational time. However this increase is not sufficient to prevent the use of complex models on data sets of usual sizes, as it takes a little bit more than an hour and a quarter to optimize parameters with the richest models on a data set containing 92 sequences. It is also noteworthy that the Bio++ implementation requires less memory than other programs. This is partly explained by differences in the algorithms used to compute the likelihood [28]. The PhyML programs, including nhPhyML, use a double-recursive algorithm [6], which saves a lot of computation when exploring the space of tree topologies but results in a three fold increase in memory usage compared to the simple-recursive algorithm. Because no tree space exploration was involved, BppML computations used the simple-recursive algorithm. If desired, however, Bio++ also offers the double-recursive algorithm.

**Table 1:** Comparison of the NHML, (NH)PhyML and BppML programs. Likelihood: - log (likelihood) of the optimized parameters, with a fixed tree topology.

Likelihood												
Rate	Constant			Γ(4)			Γ(4) + *I*			Covarion		

Model	H	NH1	NH3	H	NH1	NH3	H	NH1	NH3	H	NH1	NH3

NHML	15307	15034	--	14145	13828	--	--	--	--	13750	**13397**	--
PhyML	**15187**	15011	--	**14141**	13824	--	**14128**	--	--	--	--	--
BppML	15187	**14920**	15109	14141	**13821**	14029	14128	13810	14018	**13747**	13399	13615

Time												

Rate	Constant			Γ(4)			Γ(4) + *I*			Covarion		

Model	H	NH1	NH3	H	NH1	NH3	H	NH1	NH3	H	NH1	NH3

NHML	0:01:40	0:02:28	--	0:03:07	**00:02:13**	--	--	--	--	0:19:24	**0:19:09**	--
PhyML	**0:00:07**	**0:01:43**	--	**0:00:34**	00:02:29	--	**0:00:35**	--	--	--	--	--
BppML	0:00:27	0:11:57	0:01:12	0:00:47	00:35:46	0:00:48	0:01:01	0:29:40	0:01:38	**0:02:52**	1:14:32	0:14:27

Memory												

Rate	Constant			Γ(4)			Γ(4) + *I*			Covarion		

Model	H	NH1	NH3	H	NH1	NH3	H	NH1	NH3	H	NH1	NH3

NHML	16.38	20.48	--	55.30	65.54	--	--	--	--	55.30	65.54	--
PhyML	10.24	28.67	--	30.73	77.82	--	30.72	--	--	--	--	--
BppML	**08.19**	**08.19**	08.19	**14.34**	**14.34**	14.34	**14.34**	16.38	16.38	**12.29**	**14.34**	12.29

Time is shown as hours:minutes:seconds. Numbers in bold font correspond to the best performance for each comparison. Memory corresponds to the maximum memory usage during the program execution in megabytes. H: homogeneous case, with a K80 substitution model, NH1: theta per branch model, with a T92 substitution model, NH3: clade-specific and G+C-rich species theta model, see methods. The PhyML program was used for the H model, and nhPhyML for the NH1 model.

**Figure 4:**
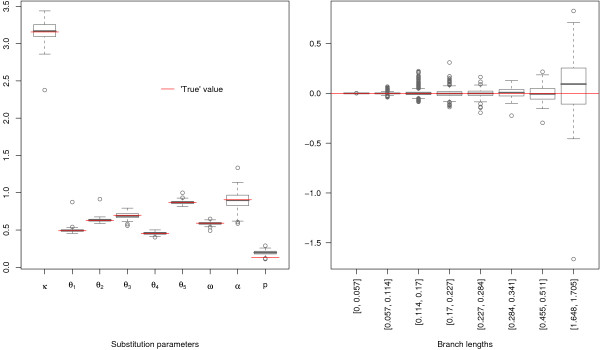
**Assessing parameter estimation using simulations**. Left: boxes show the median and quartiles of the distribution of parameter estimates for 100 simulations. The 'true' value used in the simulation is shown in red. Right: boxes show the distribution of the bias (estimated value – real value), as a function of the (pooled) real values of the branch length. θ_*_: GC content, *ω *GC content at root, *α *: shape of the Gamma distribution of rates across sites, *p *proportion of invariant sites (see text for details on the model used).

The convergence of the optimization algorithm was assessed by two methods, using the NH3 model. First, we used 100 distinct randomly chosen initial sets of parameter values and the RNA data set (see methods). We found that the estimated values obtained in each run were the same for all parameters up to the 5th decimal. Second, we simulated 100 data sets using the NH3 model with a Gamma + invariant rate distribution, with parameter values estimated from the real data set and the same number of sites. These parameters were then re-estimated for each simulated data set using random initial values. The results are displayed on figure 4, and show that the parameter values are recovered without bias and with a good precision. The only exception is the proportion of invariant sites which is slightly overestimated. These results also validate the simulation procedure.

### Example of application: parametric bootstrap and Bowker's test for non-homogeneity

As most phylogenetic reconstruction models are homogeneous, they do not properly model the evolution of homologous sequences that vary widely in their compositions. Analyzing compositionally heterogeneous data sets with homogeneous models of sequence evolution may therefore lead to incorrect inferences, provided the heterogeneity is large enough. Several tests have been developed to assess the amount of heterogeneity present in a data set (see [29] for a review).

#### Estimating the amount of compositional heterogeneity in a data set

Most commonly, a matrix is assembled that contains compositions in all characters for all sequences, and this matrix is analyzed through *χ*^2 ^statistics [29]. However, this approach usually does not distinguish between constant and variable sites, and therefore may underestimate the true amount of heterogeneity in a data set [29].

Recently, Ababneh et al. [30] re-introduced Bowker's pairwise test [31] for symmetry. Given two aligned sequences *S*_1 _and *S*_2 _on a given alphabet of size *n *and characters *x*_{1,2...*n*}_, it compares the numbers of substitutions between *x*_*i *_in *S*_1 _and *x*_*j *_in *S*_2_, {*i*,*j*} ∈ [1 : *n*], with the numbers of substitutions between *x*_*j *_in *S*_1 _and *x*_*i *_in *S*_2_. If these pairs of numbers are equal for all {*i*, *j*} ∈ [1 : *n*], the two sequences may have evolved according to two identical processes. Otherwise, the two processes were necessarily different.

Bowker's test therefore permits to assess whether compositional differences have accumulated between two sequences through non-homogeneous evolution. To apply it to more than two sequences, Rodriguez-Ezpeleta et al. [32] computed all pairwise Bowker's tests in their alignment and computed the median value; one could also have counted the number of Bowker's tests that are significant at a 5% threshold according to a *χ*^2 ^table.

However, none of these tests permit to estimate if the amount of heterogeneity that they detect in a given data set is sufficient to bias inferences made using homogeneous models, although this is likely the question an average user would like to answer.

### Assessment of the fit of evolutionary models with respect to compositional heterogeneity

Here, we describe a method to reveal the ability of evolutionary models to account for the compositional heterogeneity in a sequence alignment, which we measure using the median of all Bowker's pairwise statistics, or the number of significant Bowker's pairwise tests (in the following, we note the measure of compositional heterogeneity *h*). This method is tree-based, and uses parametric bootstrapping [10-12]. In this respect, it is similar to the method recently introduced in [13] in the Bayesian setting. Our approach requires 5 steps to estimate the fit of a model *M *to a data set *D*.

1. Compute the compositional heterogeneity measure *h *for the data set *D*.

2. Estimate the parameters of model *M *based on the data set *D *according to the Maximum Likelihood criterion.

3. Simulate a large number of data sets *D' *using the model *M *previously estimated.

4. Compute the compositional heterogeneity measure *h' *for each alignment *D'*.

5. Compare the measure *h *obtained on data set *D *to measures *h' *obtained on data sets *D'*. If *h *is outside 95% of the distribution of *h'*, the model does not properly reproduce the heterogeneity of data set *D*.

Using such an approach, any model can be compared with others with respect to their ability to handle the compositional heterogeneity of a given data set: the closest the distribution of *h' *is from *h*, the highest is the fit. Ideally, the distribution of measures *h' *obtained on the parametric bootstrap replicates of a good model should be centered around the value obtained for the real alignment *h*, with a very low variance. If one neglects potential problems linked with over-parametrization, the inferences of the best model should be preferentially trusted compared to a model that fails to account for an important feature of a data set. Overall, our approach can be used for model selection, although contrary to criteria such as AIC or BIC [28] this approach does not take into account the number of parameters; more importantly, it can also be used for estimating model adequacy.

### Application to an rRNA data set

Our approach to assess the composition-wise fit of evolutionary models to a data set was applied to an alignment containing ribosomal RNA sequences from Archaea, Bacteria and Eukaryotes [6]. First, several homogeneous and non-homogeneous models were fitted to the data set, using a Tamura 1992 model of substitution with a four classes Gamma + invariant distribution of rates across sites. Then, 10,000 artificial data sets were simulated in each case using these estimated parameters. Eventually, the real data set and the simulated data sets were compared with respect to their compositional heterogeneity: models able to simulate data sets with similar amounts of heterogeneity as the real data set appropriately account for this specific aspect of the data.

**Figure 5:**
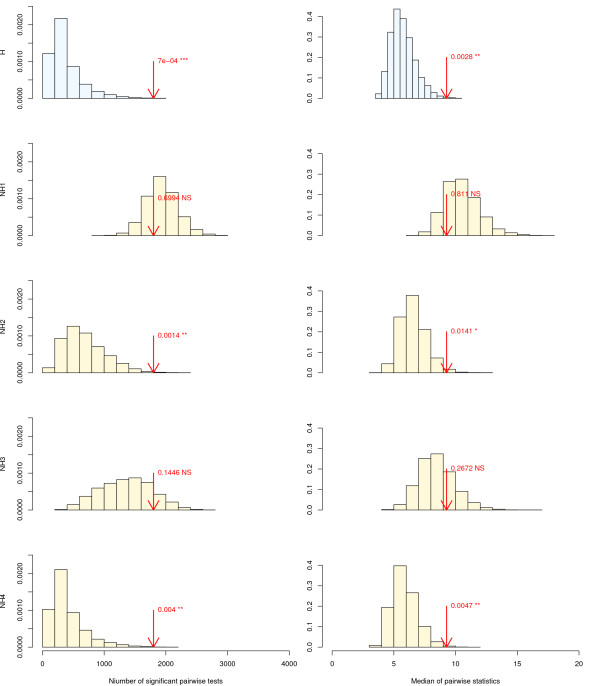
**Distributions of the Bowker's test statistics under various models**. First column: number of pairwise tests significant at the 5% level. Second column: median of the pairwise statistics. First row: homogeneous model (H). Second row: one theta per branch non-homogeneous model (NH1). Third row: 3 thetas non-homogeneous model (NH2). Fourth row: 5 thetas non-homogeneous model (NH3). Fifth row: one kappa per branch non-homogeneous model (NH4). All models use the Tamura 1992 substitution model with a 4-classes discrete Gamma + invariant rate distribution. The arrows indicate the observed values from the real data set and the resulting p-values.

Results are shown in figure 5 and table 2. Both the number of significant Bowker's tests and the median of their values give similar results. For instance, both indices find that the real data set shows significantly more heterogeneity than the distributions of data sets simulated under the homogeneous model of sequence evolution (p-value = 0.0008 for the number of significant pairwise tests and p-value = 0.0028 for the median). The homogeneous model therefore lacks parameters useful to account for this particular feature of the data. Allowing different transition/transversion rates for each branch as in model NH4 does not solve this problem, as the obtained bootstrapped distribution also significantly underestimates the heterogeneity in the real data (p-value = 0.0015 and p-value = 0.0047, respectively). It is noteworthy, however, that the likelihood ratio test finds that this model describes the data significantly better than the homogeneous one, whereas the AIC and BIC criteria do not. On the contrary, the NH1 model simulated sequences distribution surrounds the value obtained on the real data set (p-value > 0.7 in both cases). This suggests that Galtier and Gouy's modeling [24] properly accounts for the heterogeneity in rRNA data sets, and that there may be no point in using more parameter-rich models such as Yang and Roberts' [33] on these molecules. The results even suggest that NH1 might be slightly prone to over-estimating the amount of heterogeneity. For instance, the median Bowker's test value for simulated data sets are most often higher than the value obtained on the real data set. NH1's behavior may be explained by over-parametrization: it is likely that during sequence evolution, not all branches witnessed significant shifts in mutational parameters or selection pressures. To investigate further the impact of the number of parameters on model fit, two other models were tested: NH2, in which different equilibrium G+C contents are associated to each kingdom, and NH3, which further adds two equilibrium G+C contents, one for the hyperthermophilic (G+C rich) Bacteria, and one for the G+C rich Eukaryote Giardia. Hyperthermophilic (G+C rich) Archaea were not considered separately from the others as nearly all Archaea in our data set were thermophilic or hyperthermophilic. The NH2 model seems to lack useful parameters to properly account for the heterogeneity in the real data set, as its simulated data sets are less heterogeneous than the real one (p-value = 0.0040 for the number of pairwise tests, and 0.0141 for the median). The NH3 model improves upon NH2 as its bootstrapped distribution is more centered upon the observed value, which is no longer rejected (p-value = 0.14 and 0.27). However, the observed value is still on the right side of the null-distribution, and it is very likely that the correct parametrization lays between NH1, too rich with its 182 equilibrium G+C contents, and NH3, maybe too poor with its 5 equilibrium G+C contents. However, as NH3 provides a fit nearly as good as NH1 with a much lower amount of parameters, the best model may well have less than a dozen equilibrium G+C contents. Interestingly, Bowker's tests are in agreement with the Bayesian information criterion (BIC, see table 2) and favor the NH3 model. Conversely, Akaike's information criterion (AIC) and the likelihood ratio test (LRT) favor the more parameter-rich model NH1. Obviously, although a few works already addressed this issue in the Bayesian framework [11,13,34], automatic ways to explore and choose among heterogeneous models in a maximum likelihood framework are much needed. All the tools required for such a project are now available in the Bio++ libraries.

**Table 2:** Model comparisons.

Model	lnL	*k*	LRT			AIC	BIC	Bowker	
			H	NH2	NH3			# tests	median
H	-14110.628293	185				28591.26	29380.69	0.0008	0.0028
NH1	-13810.371502	368	600.51	556.74	416.97	**28356.74**	29927.07	0.7010	0.8110
NH2	-14088.739682	189	43.78			28555.48	29361.98	0.0040	0.0141
NH3	-14018.854234	191	183.55	139.77		28419.71	**29234.74**	0.1448	0.2672
NH4	-13970.841467	368	279.57			28677.68	30248.01	0.0015	0.0047

Comparison of the various non-homogeneous models with the homogeneous case, using different criteria. *k *is the number of parameters and *lnL *is the log likelihood of each model. The Akaike's information criterion (AIC) of each model is defined as 2*k *- 2·*lnL*, and the lowest value, corresponding to the best model according to this criterion is in bold font. The Bayesian information criterion (BIC) is computed as *k*· ln(*n*) - 2·*lnL*, *n *= 527 being the number of observations. The lowest value is in bold font. The likelihood ratio test (LRT) allows to compare nested models only, and is defined as minus two times the logarithm of the ratio of likelihoods. All LRT are significant at the 0.1% level. This ratio follows a *χ*^2 ^distribution with the number of additional parameters as the degrees of freedom. The last two columns show the p-values of the two Bowker's test introduced in this paper.

## Conclusion

Bio++ is a growing set of libraries designed for sequence, phylogenetic and molecular evolution analyzes. In this article extensions allowing to implement a wide variety of non-homogeneous models of sequence evolution were introduced. Combined with support for rates across sites and heterotachous models of evolution, and with routines for optimizing parameters and tree topology in the maximum likelihood framework, they provide a comprehensive platform for phylogenetic studies, either for bioinformaticians willing to develop their own software, or for biologists characterizing the evolution of a particular set of sequences using the BppML and BppSegGen programs. Whilst being a generalist program implementing a large variety of models, BppML was shown to be of a similar quality as programs dedicated to particular homogeneous or non-homogeneous models of evolution, achieving higher likelihood scores with smaller memory requirements while conserving reasonable running-times. Its joint use with BppSeqGen permits to precisely study the evolution of a particular data set through parametric bootstrapping, and may be used to generate realistic artificial data sets to study the robustness of phylogenetic reconstruction methods in the presence of heterogeneity and heterotachy. Further developments may involve methods to optimize the number of models necessary to account for the heterogeneity in a data set, or methods to explore the space of tree topologies with a broad range of non-homogeneous models of sequence evolution.

## Methods

### Data and phylogeny reconstruction

RNA sequences from the small and the large subunit of the ribosome were aligned and concatenated. Sequences coming from 22 Archaea, 34 Bacteria and 36 Eukaryotes were selected to yield a data set containing 92 sequences and 527 complete sites, with G+C contents ranging from 43% to 71%. A phylogenetic tree was built with nhPhyML [6]. For additional information, please refer to [6].

### Comparing likelihood optimizations

The NHML, (NH)PhyML and BppML programs were used to compare optimization performances. The programs were run on the data set from [6], after all columns in the alignment containing at least either a gap or an unknown character had been removed. The phylogenetic tree from [6] was used as a fixed topology, and the branch lengths used as initial values for the optimization. To allow the comparison between the three programs, the Kimura two parameters model of substitution [35] was used for homogeneous models and models derived from Tamura's 1992 model [27] for non-homogeneous models. Initial values were set to 1 and 0.5 for the *κ *and *θ *parameters respectively. A Gamma (4 classes) + invariant rates across sites distribution was also tested, with initial value set to 0.5 for the Gamma shape parameter, and 0.2 for the proportion of invariants. Galtier's 2001 [22] heterotachous model was also tested, with 4 rate classes, initial values of the shape parameter set to 0.5, and initial value of the rate change parameter set to 0.5. The precision in the optimization algorithm was set to 0.000001 for the three programs. The total length of execution was corrected according to the average CPU usage, and the memory usage corresponds to the maximum reached during program execution, as reported by the Unix "top" command. All calculations were performed on a 64 bits Intel(R) Core(TM)2 Duo, CPU 2.66 GHz.

### Assessing the convergence of the optimization procedure

Different initial values were used as initial guesses for the optimization algorithm. The GC frequencies and the proportion of invariant sites were chosen randomly from a uniform distribution between 0 and 1. The transitions/transversions ratio and the alpha parameter of the rate distribution were picked from a [0, 5] and [0.2, 2] uniform distributions, respectively. Branch lengths were taken from a uniform distribution between 0 and 0.1.

### Computing p-values for Bowker tests

Alignment-wise tests for non-homogeneity were performed using two types of statistics:

• The number of 5% significant pairwise tests,

• The median of pairwise statistics.

In both cases, the global p-value was computed as

(1) $$\text{p}-\text{value}=\frac{N_{2}+1}{N_{1}+1},$$

where *N*_1 _is the number of simulations performed under the null model, and *N*_2 _is the number of values of the statistic in the simulations that were greater or equal to the observed one, measured from the real data set. In this study, *N*_1 _was set to 10,000.

Program source code for performing Bowker's test is provided as Additional file 2. The data and scripts to run the analyses are in Additional file 3.

## Availability and requirements

**Project name: **The Bio++ libraries (version 1.6) and programs suite (version 1.0).

**Project home page: **http://kimura.univ-montp2.fr/BioPP and http://home.gna.org/bppsuite

**Operating systems: **Any platform with a C++ compiler and supporting the Standard Template Library

**Programming language: **C++

**Other requirements: **The C++ Standard Template Library

**License: **The CeCILL free software license (GNU compatible)

## Authors' contributions

BB and JD designed the method, implemented the software and wrote the article. JD ran the analyses.

## Supplementary Material

Additional file 1 **Detailed results of model comparison**. OpenDocument spreadsheet (.ods) file containing detailed results from table 1, with parameter estimates obtained. Click here for file

Additional file 2 **Program to compute Bowker's test**. Zip archive containing the C++ program used to compute Bowker's test. Click here for file

Additional file 3 **Data set, tree and scripts for running Bowker's tests**. Zip archive containing the sequence alignment and phylogenetic tree used, together with scripts for running the tests presented in this article. Click here for file

## References

[B1] Williams PD Pollock DD Blackburne BP Goldstein RA Assessing the accuracy of ancestral protein reconstruction methods PLoS Comput Biol 2006 2 e69 e69 10.1371/journal.pcbi.0020069 16789817 PMC1480538

[B2] Goldman N Statistical tests of models of DNA substitution J Mol Evol 1993 36 182 198 10.1007/BF00166252 7679448

[B3] Kuhner MK Felsenstein J A simulation comparison of phylogeny algorithms under equal and unequal evolutionary rates Mol Biol Evol 1994 11 459 468 8015439 10.1093/oxfordjournals.molbev.a040126

[B4] Guindon S Gascuel O A simple, fast, and accurate algorithm to estimate large phylogenies by maximum likelihood Syst Biol 2003 52 696 704 10.1080/10635150390235520 14530136

[B5] Lopez P Casane D Philippe H Heterotachy, an important process of protein evolution Mol Biol Evol 2002 19 1 7 11752184 10.1093/oxfordjournals.molbev.a003973

[B6] Boussau B Gouy M Efficient likelihood computations with nonreversible models of evolution Syst Biol 2006 55 756 768 10.1080/10635150600975218 17060197

[B7] Kolaczkowski B Thornton JW Performance of maximum parsimony and likelihood phylogenetics when evolution is heterogeneous Nature 2004 431 980 984 10.1038/nature02917 15496922

[B8] Philippe H Zhou Y Brinkmann H Rodrigue N Delsuc F Heterotachy and long-branch attraction in phylogenetics BMC Evol Biol 2005 5 50 50 10.1186/1471-2148-5-50 16209710 PMC1274308

[B9] Goldman N Anderson JP Rodrigo AG Likelihood-based tests of topologies in phylogenetics Syst Biol 2000 49 652 670 10.1080/106351500750049752 12116432

[B10] Bollback JP Bayesian model adequacy and choice in phylogenetics Mol Biol Evol 2002 19 1171 1180 12082136 10.1093/oxfordjournals.molbev.a004175

[B11] Foster PG Modeling compositional heterogeneity Syst Biol 2004 53 485 495 10.1080/10635150490445779 15503675

[B12] Lartillot N Brinkmann H Philippe H Suppression of long-branch attraction artefacts in the animal phylogeny using a site-heterogeneous model BMC Evol Biol 2007 7 Suppl 1 S4 S4 10.1186/1471-2148-7-S1-S4 17288577 PMC1796613

[B13] Blanquart S Lartillot N A Site- and Time-Heterogeneous Model of Amino-Acid Replacement Mol Biol Evol 2008 10.1093/molbev/msn018 18234708

[B14] Rambaut A Grassly NC Seq-Gen: an application for the Monte Carlo simulation of DNA sequence evolution along phylogenetic trees Cabios 1997 13 235 238 9183526 10.1093/bioinformatics/13.3.235

[B15] Yang Z PAML 4: phylogenetic analysis by maximum likelihood Mol Biol Evol 2007 24 1586 1591 10.1093/molbev/msm088 17483113

[B16] Sueoka N On the genetic basis of variation and heterogeneity of DNA base composition Proc Natl Acad Sci USA 1962 48 582 592 10.1073/pnas.48.4.582 13918161 PMC220819

[B17] Galtier N Lobry JR Relationships between genomic G+C content, RNA secondary structures, and optimal growth temperature in prokaryotes J Mol Evol 1997 44 632 636 10.1007/PL00006186 9169555

[B18] Foster PG Jermiin LS Hickey DA Nucleotide composition bias affects amino acid content in proteins coded by animal mitochondria J Mol Evol 1997 44 282 288 10.1007/PL00006145 9060394

[B19] Zeldovich KB Berezovsky IN Shakhnovich EI Protein and DNA sequence determinants of thermophilic adaptation PLoS Comput Biol 2007 3 e5 e5 10.1371/journal.pcbi.0030005 17222055 PMC1769408

[B20] Wang HC Spencer M Susko E Roger AJ Testing for covarion-like evolution in protein sequences Mol Biol Evol 2007 24 294 305 10.1093/molbev/msl155 17056642

[B21] Dutheil J Gaillard S Bazin E Glémin S Ranwez V Galtier N Belkhir K Bio++: a set of C++ libraries for sequence analysis, phylogenetics, molecular evolution and population genetics BMC Bioinformatics 2006 7 188 188 10.1186/1471-2105-7-188 16594991 PMC1501049

[B22] Galtier N Maximum-likelihood phylogenetic analysis under a covarion-like model Mol Biol Evol 2001 18 866 873 11319270 10.1093/oxfordjournals.molbev.a003868

[B23] Tuffley C Steel M Modeling the covarion hypothesis of nucleotide substitution Math Biosci 1998 147 63 91 10.1016/S0025-5564(97)00081-3 9401352

[B24] Galtier N Gouy M Inferring pattern and process: maximum-likelihood implementation of a nonhomogeneous model of DNA sequence evolution for phylogenetic analysis Mol Biol Evol 1998 15 871 879 9656487 10.1093/oxfordjournals.molbev.a025991

[B25] Felsenstein J PHYLIP (Phylogeny Inference Package) version 3.6 Distributed by the author 2005

[B26] Press WH Teukolsky SA Vetterling WT Flannery BP Numerical Recipes in C. The Art of Scientific Computing 1992 second Cambridge University Press

[B27] Tamura K The rate and pattern of nucleotide substitution in Drosophila mitochondrial DNA Mol Biol Evol 1992 9 814 825 1528108 10.1093/oxfordjournals.molbev.a040763

[B28] Felsenstein J Inferring Phylogenies 2004 Sinauer Associates, Inc

[B29] Jermiin L Ho SY Ababneh F Robinson J Larkum AW The biasing effect of compositional heterogeneity on phylogenetic estimates may be underestimated Syst Biol 2004 53 638 643 10.1080/10635150490468648 15371251

[B30] Ababneh F Jermiin LS Ma C Robinson J Matched-pairs tests of homogeneity with applications to homologous nucleotide sequences Bioinformatics 2006 22 1225 1231 10.1093/bioinformatics/btl064 16492684

[B31] Bowker A A test for symmetry in contingency tables J Am Stat Assoc 1948 43 572 574 10.2307/2280710 18123073

[B32] Rodríguez-Ezpeleta N Brinkmann H Roure B Lartillot N Lang BF Philippe H Detecting and overcoming systematic errors in genome-scale phylogenies Syst Biol 2007 56 389 399 10.1080/10635150701397643 17520503

[B33] Yang Z Roberts D On the Use of Nucleic Acid Sequences to Infer Branchings in the Tree of Life Mol Biol Evol 1995 12 451 458 7739387 10.1093/oxfordjournals.molbev.a040220

[B34] Blanquart S Lartillot N A Bayesian Compound Stochastic Process for Modeling Nonstationary and Nonhomogeneous Sequence Evolution Mol Biol Evol 2006 23 2058 2071 10.1093/molbev/msl091 16931538

[B35] Kimura M A simple method for estimating evolutionary rates of base substitutions through comparative studies of nucleotide sequences J Mol Evol 1980 16 111 120 10.1007/BF01731581 7463489

